# Screening and performance optimization of fungi for heavy metal adsorption in electrolytes

**DOI:** 10.3389/fmicb.2024.1371877

**Published:** 2024-03-25

**Authors:** Yuhui Yang, Rui Liu, Yizhou Zhou, Yingnan Tang, Jing Zhang, Yu Wang, Tingting Dai, Ping Zou, Xiaoyi Bi, Shuibing Li

**Affiliations:** ^1^Institute of International Rivers and Eco-Security, Yunnan University, Kunming, China; ^2^School of Ecology and Environmental Science, Institute for Ecological Research and Pollution Control of Plateau Lakes, Yunnan University, Kunming, China; ^3^Institute of Metal Research, Chinese Academy of Sciences, Shenyang, China; ^4^Key Laboratory of Pollution Ecology and Environmental Engineering, Institute of Applied Ecology, Chinese Academy of Sciences, Shenyang, China; ^5^International School of Shenyang Jianzhu University, Shenyang, China

**Keywords:** biosorption, industrial wastewater treatment, *Paecilomyces lilacinus*, precious metals recovery, superalloy electrolytes

## Abstract

The resource recovery and reuse of precious metal-laden wastewater is widely recognized as crucial for sustainable development. Superalloy electrolytes, produced through the electrolysis of superalloy scrap, contain significant quantities of precious metal ions, thereby possessing substantial potential for recovery value. This study first explores the feasibility of utilizing fungi to treat Superalloy electrolytes. Five fungi resistant to high concentrations of heavy metals in electrolytes (mainly containing Co, Cr, Mo, Re, and Ni) were screened from the soil of a mining area to evaluate their adsorption characteristics. All five fungi were identified by ITS sequencing, and among them, *Paecilomyces lilacinus* showed the best adsorption performance for the five heavy metals; therefore, we conducted further research on its adsorption characteristics. The best adsorption effect of Co, Cr, Mo, Re, and Ni was 37.09, 64.41, 47.87, 41.59, and 25.38%, respectively, under the conditions of pH 5, time 1 h, dosage 26.67 g/L, temperature 25–30°C, and an initial metal concentration that was diluted fivefold in the electrolyte. The biosorption of Co, Mo, Re, and Ni was better matched by the Langmuir model than by the Freundlich model, while Cr displayed the opposite pattern, showing that the adsorption process of *P. lilacinus* for the five heavy metals is not a single adsorption mechanism, but may involve a multi-step adsorption process. The kinetics study showed that the quasi-second-order model fitted better than the quasi-first-order model, indicating that chemical adsorption was the main adsorption process of the five heavy metals in *P. lilacinus*. Fourier transform infrared spectroscopy revealed that the relevant active groups, i.e., hydroxyl (-OH), amino (-NH_2_), amide (- CONH_2_), carbonyl (-C = O), carboxyl (-COOH), and phosphate (PO_4_^3–^), participated in the adsorption process. This study emphasized the potential application of *P. lilacinus* in the treatment of industrial wastewater with extremely complex background values.

## 1 Introduction

Superalloys are cutting-edge industrial materials with unique properties, such as excellent creep resistance, anti-corrosion, and anti-oxidation, which allows them to be used in many harsh applications, such as gas turbines in aircraft engines and power plants (Chen et al., 2023a). Superalloys are composed of numerous rare and precious metals; therefore, their production includes waste containing various precious metals, including Ni, Re, Cr, Mo, Co, W, and others (Cui et al., 2020). If directly discharged into the environment, they can be extremely harmful (Zeng et al., 2022). As these heavy metals are not biodegradable, they are easily transferred and enriched among crops, fish, and other substances, eventually reaching the human body through the food chain. When the concentration of heavy metals exceeds a certain threshold, it causes serious damage to the human body, including the respiratory, digestive, and nervous systems, and may endanger life in extreme cases (Zeng et al., 2022; Zhao et al., 2023).

Furthermore, precious metals are scarce on earth, and China is heavily dependent on imports, so they are not only expensive but also greatly influenced by external factors (Zhang et al., 2023). Therefore, the effective recovery and utilization of precious metals in such wastes is not only conducive to the comprehensive utilization of China’s metal resources, but also to reducing harm to the environment, with significant economic and environmental benefits (Xing et al., 2013). According to statistics, e-waste treatment companies in the United States alone make $25 million in annual profits. For example, it costs about $300,000 to mine a ton of silver, but only $10,000 to recover a ton of silver from waste. Superalloy electrolytes are a solution produced by the electrolysis of superalloy scrap, and contain large quantities of precious metal ions (Song et al., 2018), so they have great potential recovery value.

The effective treatment methods for mitigating heavy metals in polluted water mainly encompass chemical precipitation, electrochemical redox, ion exchange, adsorption and membrane(Qiu et al., 2021). In contrast, biosorption has the characteristics of economic efficiency, good selectivity, reusability, easy separation and recovery of metals, and no secondary pollution (Bashir et al., 2019), thus becoming a promising method for heavy metal wastewater treatment. In biosorption, the filamentous fungi used are inexpensive, survive well, and are easy to operate, which are characteristics conducive to large-scale industrial applications (Narolkar et al., 2022). At present, biosorption technologies in China and abroad are only used at the laboratory level, and the solutions used are self-formulated with low concentrations of common metals. However, this approach is still far from practically viable at an industrial scale. Superalloy electrolytes are a type of strongly acidic multi-metal mixed waste solution that is produced in actual production and has an extremely high concentration. So, it is extremely difficult to treat, and there is no research on treating superalloy electrolytes using biosorption. Therefore, we explored the adsorption of heavy metals in such electrolytes by fungi in this study.

The objectives of this study were as follows: (1) To screen fungi that are resistant to heavy metals in superalloy electrolytes and that have high adsorption performance in mining areas. (2) To explore the optimal adsorption conditions and mechanisms of the fungi to determine their adsorption characteristics, and (3) To address the paucity of biosorption technology to treat electrolytes, which are extremely difficult to treat, and provide a scientific basis for the biological treatment of actual industrial wastewater.

## 2 Materials and methods

### 2.1 Preparation of the heavy metal stock solution

The electrolyte generated by dissolving superalloy waste from an enterprise in Shenyang was used as a heavy metal stock solution. The electrolyte, which is a type of strongly acidic wastewater (pH = 2.6), mainly contained five heavy metal elements (Co, Cr, Mo, Re, and Ni) at concentrations of 10317, 4537, 35, 2557, and 39088 mg/L, respectively.

### 2.2 Isolation and identification of fungal strains

All five fungal strains were isolated from soil in the Yangjiazhangzi Mine in Huludao City (120°36′E, 40°53′N). Potato Dextrose Agar medium (200 g potato, 20 g glucose, 15 g agar, and 1000 mL water), Autotrophic Inorganic Salt medium [3.0 g (NH_4_)_2_SO_4_, 0.1 g KCl, 0.5 g K_2_HPO_4_, 0.5 g MgSO_4_⋅7H_2_O, 0.02 g NaNO_3_, 0.01 g CaCl_2_, 0.5 g FeSO4⋅7H_2_O, 15 g agar, and 1000 mL water], and Heterotrophic Inorganic Salt medium [3.0 g (NH_4_)_2_SO_4_, 2.0 g Na_2_CO_3_, 0.1 g KCl, 0.5 g K_2_HPO_4_, 0.5 g MgSO_4_⋅7H_2_O, 0.01 g Ca (NO_3_)_2_, 0.5 g FeSO_4_⋅7H_2_O, 15 g agar, and 1000 mL water] were used for screening, with 10% electrolyte added as the screening condition. In a 10% electrolyte solution, the concentrations of each heavy metal, namely Co, Cr, Mo, Re, and Ni, were 1031, 453, 3.5, 255, and 3908 mg/L, respectively. These three culture media can comprehensively cover the growth requirements of different types of microorganisms, facilitating the effective and comprehensive screening of target microorganisms that can tolerate electrolytes (Talukdar et al., 2020; Henagamage et al., 2022).

In the end, five fungi grew successfully in the culture medium containing the electrolyte, indicating that they were resistant to the heavy metals in the electrolyte. Fungal DNA was extracted using urea extraction (Sun et al., 2000). PCR amplification was performed using the universal primers ITS4 and ITS5. A 1.0% (w/v) agarose gel was prepared and the PCR products were verified by gel electrophoresis using the DNA Marker from BM2000 as a control. After successful amplification, the PCR products were sent to Tsingke Biotechnology Co., Ltd. for sequencing, and the obtained ITS sequences were compared using BLAST in the NCBI database to identify the fungal strains.

### 2.3 Screening of strains with high adsorption capacity for heavy metals in a superalloy electrolyte

The wet bodies of the five selected fungal strains were collected separately, and the fungal spores on the solid medium were inoculated into the PDA liquid medium with glass beads using an inoculation loop and incubated in a shaker (BSD-YX2200, BOXUN, China) at 28°C and 150 rpm. After 36 h of incubation, the spores were reintroduced into the PDA liquid medium at 10% inoculum and incubated at 28°C and 150 rpm. After 48 h of incubation, the fungal bodies were collected by centrifugation (5804R; Eppendorf, Germany) at 10000 rpm for 10 min and rinsed twice with sterile water. We then added 1.5 g of the wet bodies of the five selected fungal strains to 150 mL of the 10% electrolyte solution and placed in shaker (1 h, 28°C, 150 rpm) to conduct an adsorption reaction. After the adsorption reaction was complete, the concentrations of the five heavy metals in the electrolyte (Co, Cr, Mo, Re, and Ni) remaining in the supernatant of each sample were quantified after centrifugation (10000 rpm, 10 min). We found that all five heavy metals remained in the solution. Their concentrations were then quantified by ICP-AES (PlasmaQuant PQ9000, Analytik Jena AG, Germany).

The adsorption rate η and adsorption capacity *q* were used to evaluate the adsorption ability of the five fungal strains for heavy metals (Equations 1 and 2). The strain with the highest adsorption capacity was selected for further analysis:


(1) $$\eta =\frac{{\text{C}}_{0}-{\text{C}}_{\text{e}}}{{\text{C}}_{0}}\times 100\%$$

(2) $$\text{q}=\frac{\left({\text{C}}_{0}-{\text{C}}_{\text{e}}\right)\,V}{\text{m}}$$

*C*_0_ – concentration of heavy metals in the electrolyte before adsorption (mg/L).
*C*_*e*_ – concentration of heavy metals in the supernatant after adsorption (mg/L).
V – volume of the solution (L).
m – the mass of the adsorbent (g).

### 2.4 Effect of different factors on the adsorption effect of five heavy metals

In section “2.3 Screening of strains with high adsorption capacity for heavy metals in a superalloy electrolyte,” we selected the fungus with the best adsorption capacity of the five heavy metals in the electrolyte (Co, Cr, Mo, Re, Ni) as the object for further research. The initial dosage was set at 1.5 g, and the initial conditions for the biosorption process in the shaker were 25°C and 150 rpm. In 150 mL of the 10% electrolyte solution, we studied the effects of the selected fungus’ biosorption of the five heavy metals (Co, Cr, Mo, Re, and Ni) at different pH values, initial concentration, adsorption times, adsorption dosages, and adsorption temperatures: (1) To study the effect of pH (3, 4, 5, 6, 7). (2) To investigate the effect of initial electrolyte concentration, the electrolyte solution was diluted 0, 5, 10, 20, and 50-fold respectively. The pH was adjusted to the optimized conditions based on (1) above. (3) To study the effect of adsorption time (0.1, 0.5, 1, 2, 4, 8, 12, 16, 20, and 24 h), the pH and initial electrolyte concentration was adjusted to the optimized conditions based on (1) and (2) above. (4) To investigate the effect of adsorbent dosage (1.5, 2.0 2.5, 3.0, 3.5, 4.0, and 4.5 g), the pH and adsorption time were adjusted to the optimized conditions based on (1), (2), and (3) above. (5) To determine the effect of adsorption temperature (15, 20, 25, 30, 35°C), the pH, initial concentration, adsorption time, and adsorption dosage were adjusted to the optimal conditions based on the above tests, The calculation method for the adsorption effect of all samples was similar to that described in section “2.3 Screening of strains with high adsorption capacity for heavy metals in a superalloy electrolyte.”

### 2.5 Isotherm studies

During the biosorption of heavy metal ions, biosorption enters a state of equilibrium, which can be regarded as the equilibrium state of the metal adsorption on the adsorbent and the residual metal ions in the solution. Langmuir and Freundlich adsorption isotherms have often been used in related studies (Huang et al., 2020). We diluted the original electrolyte 2, 4, 6, 8, 10, 15, 20, 30, and 40 times. We used the fungus selected in section “2.3 Screening of strains with high adsorption capacity for heavy metals in a superalloy electrolyte” in 150 mL electrolyte solution under its optimal adsorption conditions, determined in section “2.4 Effect of different factors on the adsorption effect of five heavy metals.” The concentrations of heavy metal ions (Co, Cr, Mo, Re, and Ni) adsorbed onto the fungal cells at different electrolyte concentrations were determined. Langmuir and Freundlich equations were applied according to the approach of Tran and Chao (2018).

### 2.6 Kinetics studies

Kinetics studies were conducted using the selected fungus (in section “2.3 Screening of strains with high adsorption capacity for heavy metals in a superalloy electrolyte”) in 150 mL electrolyte under its optimal adsorption conditions (see section “2.4 Effect of different factors on the adsorption effect of five heavy metals”). The concentrations of heavy metal ions (Co, Cr, Mo, Re, and Ni) adsorbed onto fungal cells at 1, 5, 10, 20, 30, 60, 120, 240, 360, 480, 600, and 720 min were determined. Quasi-first-order and quasi-second-order kinetics models were applied according to Dai et al. (2020) and Rohit et al. (2021).

### 2.7 Infrared spectrum analysis

The wet fungal cells selected in section “2.4 Effect of different factors on the adsorption effect of five heavy metals” with the best adsorption before and after the adsorption test were used for the drying treatment. The wet cells before and after adsorption with the KBr powder were mixed, ground evenly in an agate mortar, pressed into thin slices, and scanned using a Nicolet iS 10 infrared spectrometer.

## 3 Results and discussion

### 3.1 Screening and identification of fungi resistant to superalloy electrolytes

**FIGURE 1:**
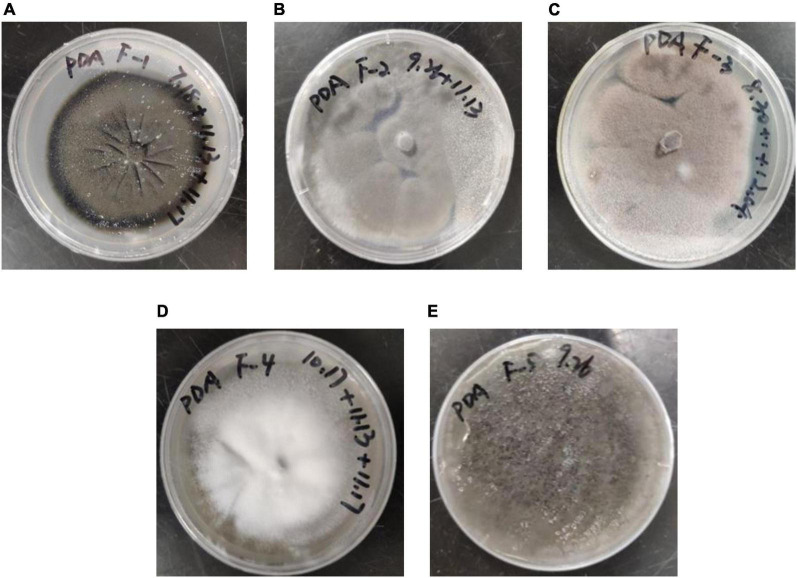
Morphological characteristics of the five screened fungal strains: **(A)** F–1, **(B)** F–2, **(C)** F–3, **(D)** F–4 and **(E)** F–5.

Five fungal strains were obtained through screening and named F-1, F-2, F-3, F-4, and F-5. The morphology of the fungi grown on PDA solid medium for 10 days is shown in Figure 1. The diameter of the F-1 colony was approximately 55 mm; it was dark green, the edge was black, the back was dark green, and the mycelia were tightly felt with concentric rings. The diameter of the F-2 colony was approximately 80 mm; it was flat, fluffy gray-green, and the back was light yellow. The diameter of the F-3 colony was approximately 70 mm; it was rose-gray, fluffy, and raised, and the back was light yellow. The diameter of the F-4 colony was approximately 60 mm; it was white, thick, hairy, raised with a dense structure, and the back was golden-yellow. The diameter of the F-5 colony was approximately 85 mm; it was dark green flocculent, the color darkened after maturity, and the back was dark green.

The internal transcribed spacer (ITS) sequences obtained from the sequencing of the five fungal strains were compared by BLAST using the GenBank database. The results of the strain sequence comparison and identification are listed in Table 1. Based on morphological characteristics and sequence homology comparison, the five fungal strains, F-1, F-2, F-3, F-4, and F-5, were tentatively identified as *Cladosporium subuliforme*, *Penicillium solitum*, *Paecilomyces lilacinus*, *Simplicillium cylindrosporum*, and *Aspergillus fumigatus*, respectively.

**TABLE 1:** Identification of five fungal strains isolated from soil in the Yangjiazhangzi Mine.

Strain code	Strain name	Amplified sequences	Similarity %
F-1	*Cladosporium subuliforme*	ITS	100
F-2	*Penicillium solitum*	ITS	99.81
F-3	*Paecilomyces lilacinus*	ITS	100
F-4	*Simplicillium cylindrosporum*	ITS	98.52
F-5	*Aspergillus fumigatus*	ITS	100

### 3.2 Adsorption capacity of five fungal strains on heavy metals in a superalloy electrolyte

Table 2 shows that among the five fungi, the highest dry matter density was recorded for *Penicillium solitum* (23.76 g/L), followed by *Simplicillium cylindrosporum* and *Paecilomyces lilacinus* at 23.12 and 22.05 g/L, respectively. The maximum biomass was observed for *Simplicillium cylindrosporum* (1.29 g/L), followed by *Penicillium solitum* and *Paecilomyces lilacinus* at 0.61 and 0.49 g/L, respectively.

**TABLE 2:** Moisture content, density and biomass of five fungal strains isolated from soil in the Yangjiazhangzi Mine.

Strain name	Moisture (%)	Dry weight (mg)	Diameter (mm)	Dry matter density (g/L)	Biomass (g/L)
*Cladosporium subuliforme*	97.89 ± 2.00	0.21 ± 0.036	2.60 ± 0.19	11.95 ± 1.36	0.29 ± 0.031
*Penicillium solitum*	96.95 ± 1.39	0.22 ± 0.033	2.10 ± 0.24	23.76 ± 0.66	0.61 ± 0.044
*Paecilomyces lilacinus*	98.11 ± 1.98	0.19 ± 0.031	2.05 ± 0.33	22.05 ± 0.74	0.49 ± 0.038
*Simplicillium cylindrosporum*	92.36 ± 2.02	0.34 ± 0.034	2.45 ± 0.26	23.12 ± 0.92	1.29 ± 0.039
*Aspergillus fumigatus*	98.02 ± 2.31	0.20 ± 0.033	2.40 ± 0.29	14.47 ± 0.95	0.37 ± 0.031

**FIGURE 2:**
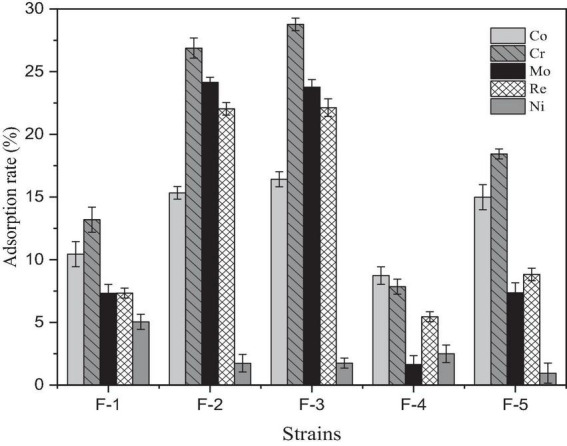
Adsorption effect of five fungal strains isolated from soil in the Yangjiazhangzi Mine on heavy metals.

*Paecilomyces lilacinus* (F-3) had the best adsorption effect on Co, Cr, Mo, and Re, with adsorption rates of 16.42, 28.77, 24.77, and 22.13%, respectively (Figure 2). However, its adsorption rate of Ni was poor.

Heavy metal adsorption capacity was calculated using moisture content, adsorption efficiency, and pre- and post-adsorption concentrations of heavy metals across the five fungal strains. Overall, *Paecilomyces lilacinus* had the highest adsorption capacities for Co, Cr, Mo, and Re, with 764.06, 0.33, 0.26, and 249.36 mg/g of dry matter, respectively. Therefore, *P. lilacinus* was selected as the biological adsorbent for the subsequent adsorption experiments.

Many studies have indicated that *P. lilacinus* possesses broad-spectrum resistance to heavy metals, indicating that it is an excellent microbial resource for remediating heavy metal pollution (Zeng et al., 2010) found that *P. lilacinus* showed resistance to Cd, Zn, Mn, Cu, Pb, and Co, and that the Cd biosorption capacity of the fungus reached 24.23 mg/g during growth. A novel microbial nanocomposite, *Paecilomyces lilacinus*—silica nanoparticle-calcium-alginate beads, has shown excellent removal efficiency of Pb (II) in aqueous solution (85.54%) at an initial concentration of 200 mg/L (Ruan et al., 2020). *Paecilomyces lilacinus* demonstrates notable efficacy in the adsorption of Cd (Chen et al., 2023b) and may be used as an inexpensive, environmentally friendly, and effective bioremediation agent to remove Cd^2+^ from wastewater (Xia et al., 2015). In this study, the electrolyte containing a high concentration of heavy metals was a complex system. Nonetheless, *P. lilacinus* exhibited tolerance to the electrolyte and demonstrated a high adsorption capacity for Co, Re, and Ni, reaching 764, 358, and 315 mg/g of dry matter (Table 3), respectively, indicating that it has considerable potential as a bioremediation strain for superalloy electrolytes.

**TABLE 3:** Adsorption capacity of heavy metals by five fungal strains isolated from soil in the Yangjiazhangzi Mine.

Strain name	Co (mg/g)	Cr (mg/g)	Mo (mg/g)	Re (mg/g)	Ni (mg/g)
*Cladosporium subuliforme*	435.27 ± 42.11	0.14 ± 0.00	0.07 ± 0.00	74.07 ± 13.28	813.28 ± 62.89
*Penicillium solitum*	442.07 ± 21.75	0.20 ± 0.01	0.16 ± 0.01	153.84 ± 21.97	194.17 ± 24.55
*Paecilomyces lilacinus*	764.06 ± 60.38	0.33 ± 0.00	0.26 ± 0.01	249.36 ± 44.17	315.39 ± 36.19
*Simplicillium cylindrosporum*	100.58 ± 12.09	0.02 ± 0.01	0.01 ± 0.00	15.20 ± 2.36	11.35 ± 19.41
*Aspergillus fumigatus*	665.81 ± 38.14	0.20 ± 0.01	0.08 ± 0.00	94.87 ± 21.78	163.73 ± 23.34

### 3.3 Effect of different factors on the adsorption of heavy metal ions by fungi

**FIGURE 3:**
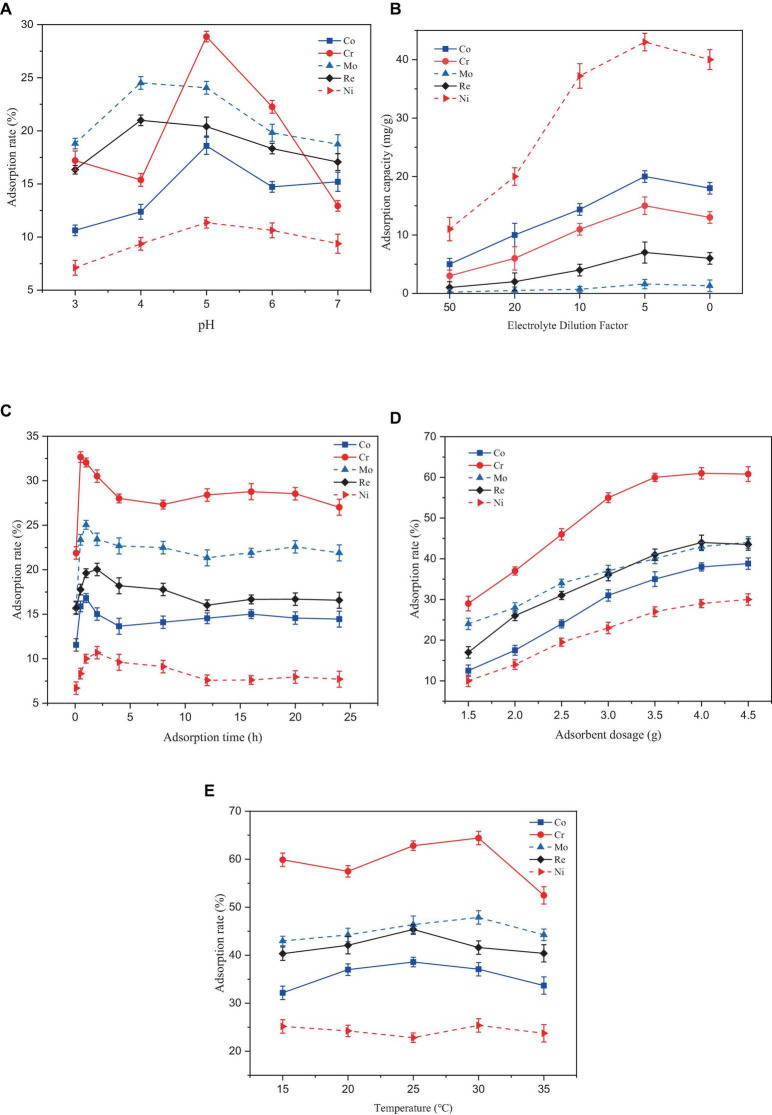
Effect of different factors on the adsorption rate of heavy metal ions by *Paecilomyces lilacinus*: **(A)** Effect of adsorption pH on adsorption efficiency, **(B)** Effect of initial concentration on adsorption efficiency, **(C)** Effect of adsorption time on adsorption efficiency, **(D)** Effect of adsorbent dosage on adsorption efficiency, **(E)** Effect of adsorption temperature on adsorption efficiency.

In Figure 3A, At a pH range of 3–7, the adsorption rate of the five heavy metals by *Paecilomyces lilacinus* showed a trend of increasing and then decreasing. The adsorption rates of Co, Cr, and Ni by *Paecilomyces lilacinus* increased rapidly with increasing pH at pH 3–5, and reached a maximum of 18.58%, 28.87%, and 11.35% at pH 5, respectively, decreasing gradually at pH – 5. The adsorption rates of Mo and Re increased rapidly at pH 3–4 and reached a maximum of 24.50 and 20.99% at a pH of 4, respectively, whereas the adsorption rate at a pH of 5 was similar to that at a pH of 4 decreasing gradually at pH > 5. Therefore, the optimal adsorption pH for heavy metals in the superalloy electrolyte is 5. In general, at pH < 7, cobalt is mainly present as Co^2+^ (Hayrettin et al., 2008). Hexavalent chromium exists mainly as HCrO^4–^, Cr_2_O7^2–^, and CrO_4_^2–^ in acidic aqueous solutions (Tandon et al., 1984) and is always negatively charged (Castro-Rodriguez et al., 2015). The two main ionic forms of molybdenum are molybdate (MoO_4_^2–^) and tetrathiomolybdate (MoS_4_^2–^) (Xu et al., 2006). Rhenium is stable in solution as ReO_4_^–^ (Srivastava et al., 2015). Under acidic conditions, the concentration of H_3_O^+^ in the solution increases, and the surfaces of suspended microorganisms can be covered by H_3_O^+^. Due to electrostatic repulsion, it is difficult for metal ions to bind to adsorption sites on the microbial surface (Mengmei and Shuliang, 2018). The repulsive force may lead to a reduced adsorption of Co and Ni on *Paecilomyces lilacinus*, but the increased electrostatic adsorption may enhance the adsorption effect of Mo and Re. The poor adsorption effect of Cr at pH = 4 may be due to the competitive adsorption between Mo, Re, and Cr. Competitive adsorption commonly occurs in multi-metal coexistence systems for the variations in the metal characteristics, electronegativity, the resultant affinity for sorption sites and the hydrated radius among metals (Lee and Chang, 2011; Park et al., 2016; Huang et al., 2018). The initial solution pH affects the degree of protonation of functional groups on the adsorbent surface. At low pH values, the protonation of the surface functional groups positively charges the surface of the adsorbent. With increasing pH, the number of negatively charged functional groups on the surface of the adsorbent gradually increases (Wen et al., 2024). Therefore, the adsorption capacity of Co and Ni gradually increased, but this was not conducive to the adsorption of Cr, Mo, and Re, so their adsorption rates decreased. When the pH continued to increase, metallic Co and Ni formed hydroxide precipitates, which were not conducive to adsorption by *P. lilacinus*.

In Figure 3B, the adsorption capacities for the five heavy metals (Co, Cr, Mo, Re, and Ni) increased with increasing initial concentration of the electrolyte. After reaching a certain value, the adsorption capacity decreased. The optimal adsorption performance was achieved when the electrolyte was diluted by a factor of 5. Generally, as the initial concentration of heavy metals in a solution increases, the adsorption capacity of the adsorbent increases until it reaches the maximum point of adsorption site saturation (Ahsaine et al., 2017). Beyond this point, further increases in the metal concentration may not result in an increased adsorption capacity and may even cause a decrease. The reasons for this phenomenon may include site saturation or electrostatic repulsion between metal ions (Ong et al., 2018) and the toxicity of high metal concentrations to microorganisms (Karthik et al., 2017).

In Figure 3C, Removal of the five heavy metals by *P. lilacinus* first increased rapidly and then slowly, reaching an adsorption dynamic equilibrium state. During the first 30 min of the reaction, *P. lilacinus* had a large number of binding sites on the surface, and the adsorption rate increased significantly, after which the rate slowed down. The adsorption rate of *P. lilacinus* to Cr peaked at 32.66% when the adsorption time was 30 min. In contrast, the adsorption rates of Co (16.82%) and Mo (25.04%) were the highest at an adsorption time of 1 h, whereas the adsorption rates of Re (20.03%) and Ni (10.68%) were highest when the adsorption time was 2 h. The reaction time after 2 h was considered the dynamic adsorption equilibrium time. Thereafter, *P. lilacinus* could no longer provide sufficient adsorption sites. Consequently, the adsorption reached saturation and the removal rate fluctuated and decreased to a certain extent. Therefore, based on the adsorption status of the five heavy metals, the ideal adsorption time of *P. lilacinus* for heavy metals in the electrolyte is 1 h.

In Figure 3D, When the dosage of *P. lilacinus* was 1.5 g, the adsorption rates of Co, Cr, Mo, Re, and Ni were 12.42, 29.45, 24.05, 17.94, and 10.11%, respectively. With an increase in the dosage, the adsorption rate of *P. lilacinus* for various metals increased linearly. The adsorption rates of Co, Cr, Mo, Re, and Ni were 40.16, 62.38, 44.91, 46.89, and 31.76%, respectively, when the dosage of *P. lilacinus* was 4 g. The removal rate did not increase significantly as the fungal dosage continued to increase. With an increased dosage of *P. lilacinus*, more surface area was available for adsorption owing to the increase in active sites on the adsorbent (Goh et al., 2019), resulting in the adsorption rate continuing to increase. However, beyond a certain range, metal adsorption reaches a saturation state (Usman et al., 2016), which may leads to a decline in the adsorption capacity for solute availability, electrostatic interaction, andinterference between the binding site (Wu et al., 2019). This phenomenon can also be explained by the “mass effect,” whereby the aggregation of abundant biosorbents reduces the effective surface area, resulting in insufficient functional sites on the cell surface for bonding metal ions and a decrease in biosorption capacity (Xia et al., 2015). Therefore, from the perspective of the comprehensive removal rate and treatment cost, the optimal dosage of *P. lilacinus* for treating 150 mL of electrolyte was 4 g, i.e., 26.67 g/L.

In Figure 3E, The adsorption temperature increased continuously in the range of 15–35°C, and the adsorption of the five heavy metals by *P. lilacinus* first increased slowly and then decreased slowly. When the adsorption temperature was 25°C, the adsorption rate of *P. lilacinus* to Co (38.58%) and Re (45.36%) peaked. When the adsorption temperature was 30°C, the adsorption rates of Cr (64.41%), Mo (47.87%), and Ni (25.38%) were at their highest. Above 30°C, the adsorption rate decreased, possibly because the increased temperature may have affected the metabolic activity of *P. lilacinus*. Microbial metabolic activity increases with increasing temperature up to an optimum level. However, excessive heat can lead to enzyme denaturation, diminished microbial activity, and potentially cause organismal inactivity or death (Thomson et al., 2017). Furthermore, temperatures exceeding the optimum temperature may induce changes in the ribosomal conformation, leading to decreased protein synthesis (Sanchez Sanchez de Groot and Ventura, 2006). It has also been reported that at high temperatures, the rate of interaction between biomass and heavy metal ions decreases. With increasing temperature, the boundary layer diminishes, resulting in a decreased removal rate of heavy metal ions (Sharma et al., 2023). In general, the best adsorption temperature range of *P. lilacinus* for heavy metals in electrolytes is 25–30°C. This may be due to the maximum activity of microbial enzymes under optimal mesophilic conditions (Jacob et al., 2018).

Based on the above conclusion, under the conditions of adsorption pH = 5, adsorption time 1 h, dosage 26.67 g/L, and adsorption temperature 25–30°C, the adsorption rates of *P. lilacinus* for Co, Cr, Mo, Re, Ni in the electrolyte were 37.09, 64.41, 47.87, 41.59, and 25.38%, respectively. Various studies have consistently observed that the optimal conditions for fungal adsorption of heavy metals, such as Co (II) (El-Bondkly and El-Gendy, 2022), Ni (II) (Sharma et al., 2021), and Cr (II) (Boujelben et al., 2022), commonly involve a pH around 5.0. Additionally, the temperature range of 25–30°C to be favorable for the maximum uptake of these heavy metals by different fungal strains (Bourzama et al., 2020; Nguyen Van et al., 2021; Kerga et al., 2023). These conclusions are consistent with the findings of the present study, indicating that these conditions could be considered optimal for the fungal adsorption of heavy metals.

### 3.4 Adsorption isotherm model

#### 3.4.1 The Langmuir isotherm model

**FIGURE 4:**
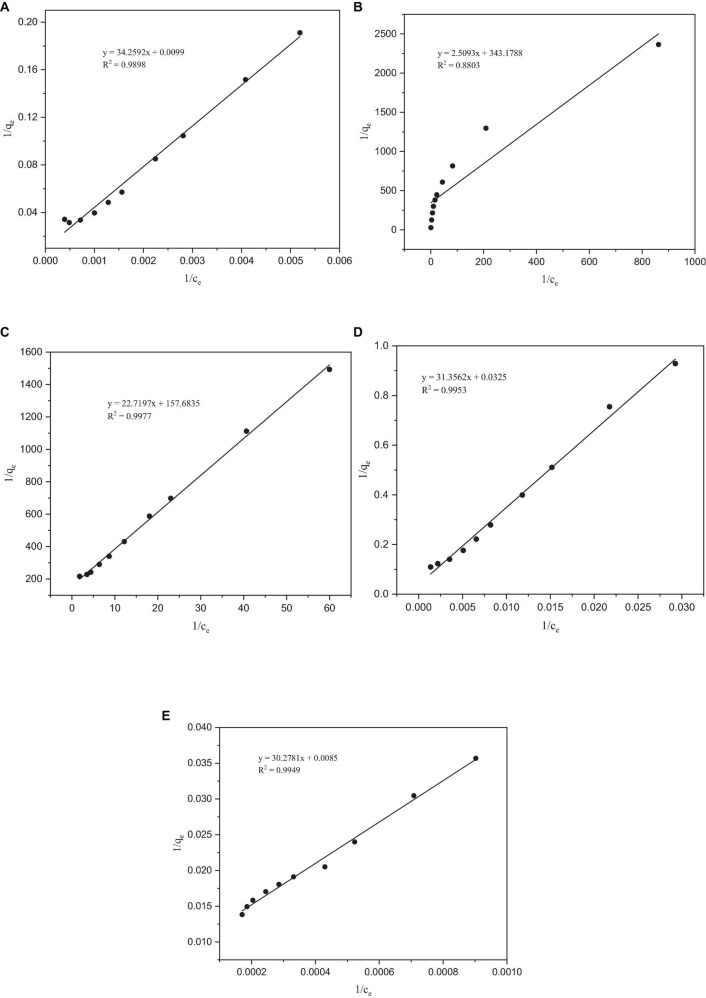
Langmuir adsorption isotherms of heavy metals by *Paecilomyces lilacinus*: **(A)** Co, **(B)** Cr, **(C)** Mo, **(D)** Re, and **(E)** Ni.

After fitting with the Langmuir isotherm model, it was found that the correlation coefficient R^2^ of the metals Co, Mo, Re, and Ni was > 0.98, except for Cr (0.88), which indicates that the Langmuir isotherm model describes the adsorption process of *P. lilacinus* to Co, Mo, Re, and Ni in the electrolyte well (Figure 4). The adsorption processes for these four heavy metals involve monolayer adsorption (the adsorbed layer is one molecule thick) on a homogeneous surface with identical active sites (Jiao et al., 2017). Adsorption is restricted to a finite number of specific sites, each of which is identical and equivalent, without any lateral interactions or steric hindrance between adjacent molecules (Vijayaraghavan et al., 2006). In its derivation, the Langmuir isotherm assumes homogeneous adsorption, wherein each molecule exhibits constant enthalpy and sorption activation energy, and all sites possess equal affinity for the adsorbent (Kundu and Gupta, 2006).

#### 3.4.2 The Freundlich isotherm model

**FIGURE 5:**
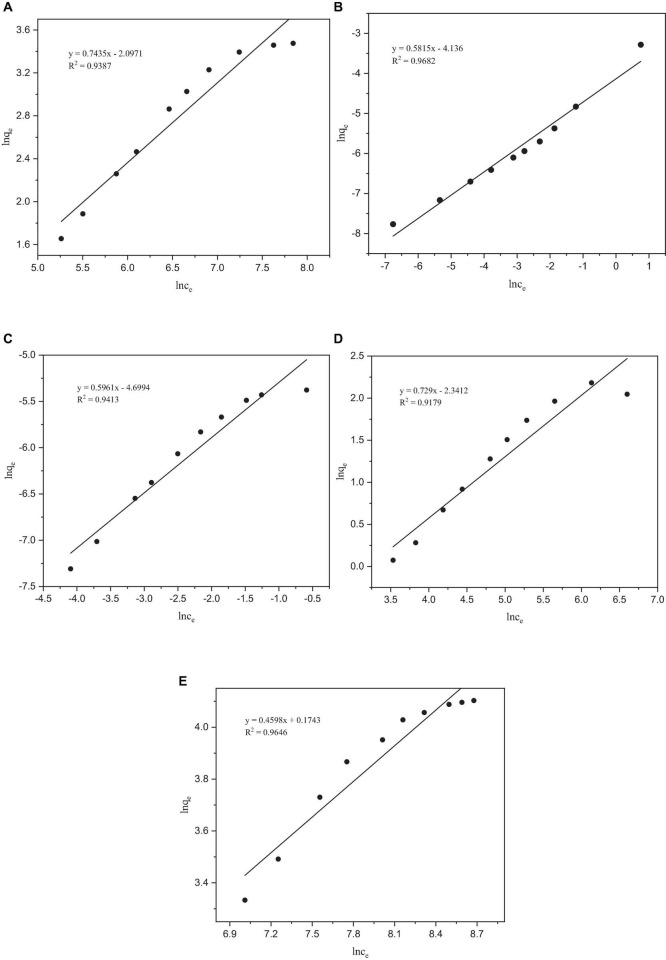
Freundlich adsorption isotherms of heavy metals by *Paecilomyces lilacinus*: **(A)** Co, **(B)** Cr, **(C)** Mo, **(D)** Re, and **(E)** Ni.

After fitting, the *R*^2^ values of the Freundlich isotherm model for Co, Mo, Re, and Ni were all lower than those of the Langmuir model (Figure 5). However, the *R*^2^ of the Freundlich isotherm model of Cr was greater than that of the Langmuir model, indicating that the adsorption of Cr occurs on a heterogeneous adsorbent surface with active sites with different adsorption energies (Duan and Fedler, 2021). The Freundlich model is applicable to adsorption across multiple layers and exhibits a non-uniform distribution of adsorption heat and affinities across the heterogeneous surface (Foo and Hameed, 2010). It is presumed that more potent binding sites are initially occupied, with the binding strength diminishing as the degree of site occupancy increases (Vijayaraghavan et al., 2006). The parameter 1/n in the Freundlich isotherm is indicative of the adsorption strength, where a value less than 1 signifies chemisorption adsorption, whereas a value higher than 1 indicates cooperative adsorption (Peckova et al., 2021). In our experiments, the fitting results for all the metals indicated chemisorption adsorption, and no cooperative adsorption was observed. Analysis of the adsorption isotherm model of *P. lilacinus* for the five target heavy metals showed that the adsorption process in the electrolyte involved a single adsorption mechanism, which may involve the occurrence of a multi-step adsorption process. The parameters of the Langmuir and Freundlich adsorption models are listed in Table 4.

**TABLE 4:** Parameters of the Langmuir and Freundlich adsorption isotherms.

	Langmuir			Freundlich		
	**q_max_ (mg/g)**	**b (L/mg)**	***R* ^2^**	**k_f_**	***n***	***R* ^2^**
Co	100.6036	0.0003	0.9898	0.1228	1.3450	0.9387
Cr	0.0029	136.7628	0.8803	0.0160	1.7198	0.9682
Mo	0.0063	6.9404	0.9977	0.0091	1.6777	0.9413
Re	30.7692	0.0010	0.9953	0.0962	1.3717	0.9179
Ni	117.9245	0.0003	0.9949	1.1904	2.1749	0.9646

It can be seen from Table 4 that the theoretical maximum adsorption capacity of Co, Cr, Mo, Re, and Ni are 100.6036, 0.0029, 0.0063, 30.7692, and 117.9245 mg/g, respectively. When the concentration of the metal ions increased, the adsorption capacity was significantly enhanced (Wu et al., 2019). Donmez and Aksu (2002) found that when the initial chromium (VI) concentration increased from 50 to approximately 250 mg/L, the adsorption capacity increased from 37.7 to 102.5 mg/g. The concentrations of Co, Cr, Mo, Re, and Ni in the electrolyte were 10317, 4537, 35, 2557, and 39088 mg/L, respectively. Therefore, it can be inferred that the main reason for the large differences in the theoretical maximum adsorption capacities of these five heavy metals may be their different initial concentrations.

### 3.5 Adsorption kinetics study

#### 3.5.1 The Pseudo-first-order kinetic model

Due to the low correlation coefficient, the *R*^2^ of each metal was below 0.6, and the fitted q_*e*_ values did not match the experimental values, indicating that the quasi-first-order kinetic model was not suitable for the adsorption of Co, Cr, Mo, Re, and Ni by *P. lilacinus*. Therefore, the fitting data graph is not listed here; only the fitting parameters of the quasi-first-order kinetics are listed in Table 5.

**TABLE 5:** Parameters of the pseudo-first-order and the pseudo-second-order kinetic models.

Pseudo-first-order kinetic					Pseudo-second-order kinetic		
	**Q (actual value) (mg/g)**	**q_e_ (due value) (mg/g)**	**k_1_ k_1_ (/min)**	***R* ^2^**	**q_e_ (due value) (mg/g)**	**k_2_ k_2_** **[g⋅(mg/min)]**	***R* ^2^**
Co	17.8932	3.6324	0.0056	0.5562	17.0155	0.0292	0.9998
Cr	0.0026	0.0004	0.0057	0.3969	0.0025	396.5542	0.9998
Mo	0.0031	0.0003	0.0007	0.1063	0.0028	−99.2140	0.9986
Re	3.6282	0.4317	0.0041	0.2515	3.4393	−0.3087	0.9995
Ni	56.1509	9.8478	0.0029	0.0523	55.2765	−0.0068	0.9985

#### 3.5.2 Pseudo-second-order kinetic model

After fitting, the correlation coefficient *R*^2^ of the quasi-second-order kinetic models of Co, Cr, Mo, Re, and Ni were all > 0.99, indicating that this model fitted the adsorption process of these five heavy metals by *P. lilacinus* very well. Under the quasi-second-order kinetic model, the *q*_*e*_ values of *P. lilacinus* to Co, Cr, Mo, Re, and Ni were 17.0155, 0.0025, 0.0028, 3.4393, and 55.2765 mg/g, respectively, which were very close to the experimental values. The fitting effect of the quasi-second-order kinetic adsorption model was better than that of the quasi-first-order kinetic model, indicating that the adsorption process of *P. lilacinus* for these five heavy metals in the electrolyte was mainly chemical adsorption, involving electron exchange or sharing with ions and the adsorbent (Huang et al., 2019).

### 3.6 Infrared spectrum

**FIGURE 6:**
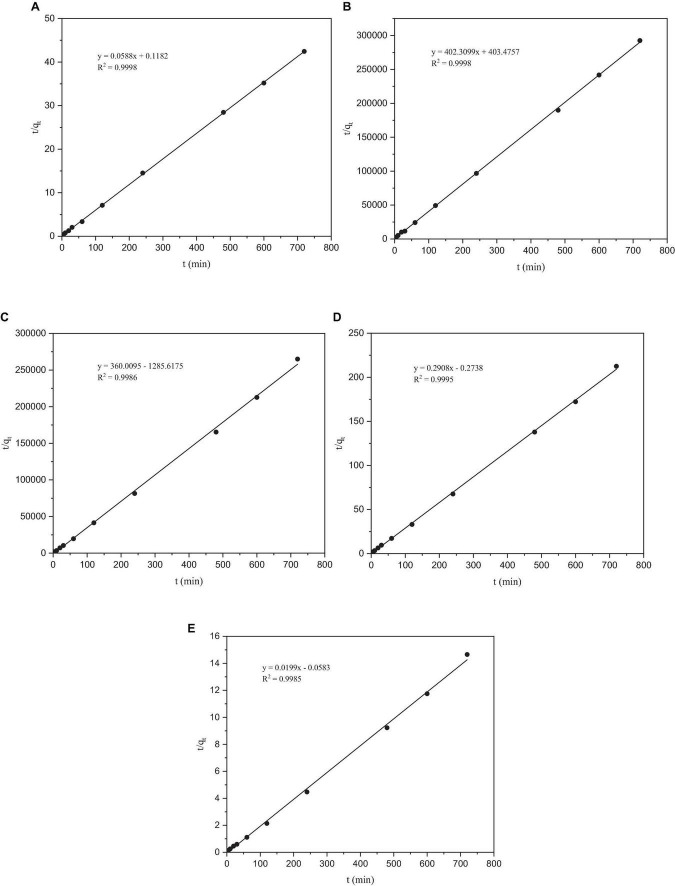
The pseudo-second-order kinetic model of heavy metals by *Paecilomyces lilacinus*: **(A)** Co, **(B)** Cr, **(C)** Mo, **(D)** Re, and **(E)** Ni.

The FTIR spectra of *P. lilacinus* before and after biosorption are shown in Figure 6. The main functional groups related to the strong peaks observed have been identified in previous studies (Zhang et al., 2011; Chen and Wang, 2016; Chen et al., 2020) and are listed in Table 6.

**TABLE 6:** Main functional groups of Fourier transform infrared spectroscopy (FTIR) analysis.

Wavelength (cm^–1^)	Functional group
3300–3400	the joint action of O-H and N-H stretching vibration
2925	C-H stretching vibration of aliphatic CH_2_
1640	C = O stretching vibration of amide I band
1545	C-N stretching vibration
1240	N-H bending vibration of amide II and amide III
1078	C-N stretching vibration in amine group C-O stretching vibration in the sugar ring the absorption peak formed by phosphate ester
500–700	The absorption peak of PO_4_^3–^

**FIGURE 7:**
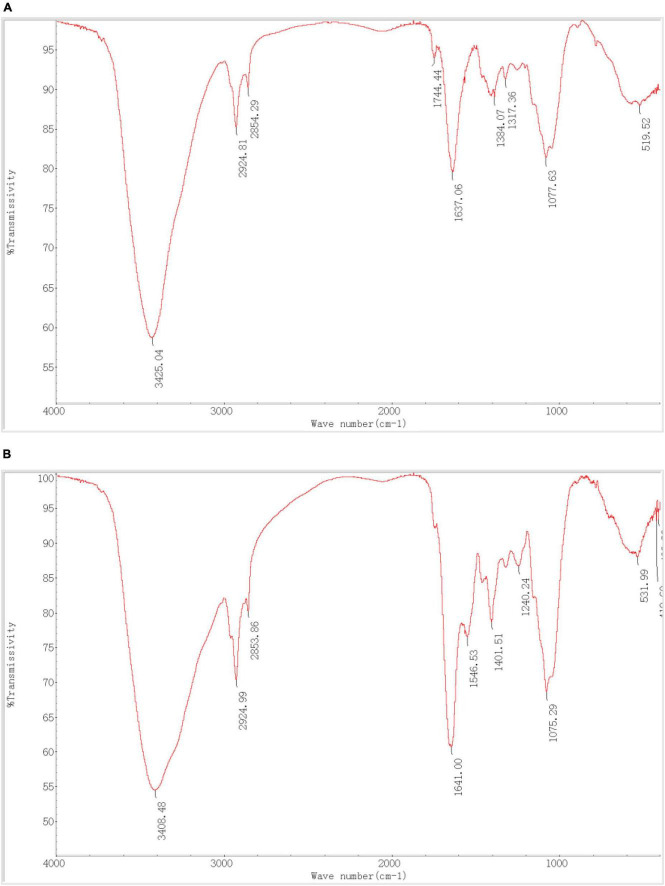
Fourier transform infrared spectroscopy (FTIR) **(A)** before and **(B)** after *Paecilomyces lilacinus* absorbed heavy metals.

As shown in Table 6, *P. lilacinus* contains–OH,–NH,–CH,–C = O, C-N, PO_4_^3–^, and other active groups. Figures 7A, B shows that the broad stretching bands at 3408.48 and 531.99 cm^–1^ strongly shifted to 3425.04 and 519.52 cm^–1^ after heavy metals loaded onto *P. lilacinus*. The other three peaks at 2924.99, 1641.00 and 1075.29 cm^–1^ are observed to become sharper after heavy-metal loading. These changes in the absorbance peaks correspond to hydroxyl (-OH), amino (-NH_2_), amide (-CONH_2_), carbonyl (-C = O), and phosphate (PO_4_^3–^) groups, which could be active functional sites (Xia et al., 2015). The bands at 1546.53 and 1240.24 cm^–1^ disappeared and the peaks at 1744.44 cm^–1^ only appeared in the spectra of metals-loaded biomass. These results indicate the presence of interactions between Co, Cr, Mo, Re, Ni, carboxyl (-COOH), and amide groups. Thus, the FTIR spectral results suggest that the five aforementioned groups on the surface of *P. lilacinus* were significant functional sites for binding heavy metal ions.

Yildirim et al. (2020) utilized *Pleurotus ostreatus* powder for Ni (II) adsorption and observed a redshift in the absorption peak of -OH (-NH) from 3275 to 3369 cm^–1^, consistent with our findings. Ou employed *Paecilomyces lilacinus* for Ag (I) adsorption and detected redshifts in the -OH peak from 3297 to 3304 cm^–1^, blueshifts in the -CH peak from 2928 to 2926 cm^–1^ and in the -C = O peak from 1653 to 1652 cm^–1^ (Ou et al., 2011). These alterations in functional groups align with our study’s findings. It was confirmed that functional groups such as -OH, -NH_2_, -COOH, and PO_4_^3–^ are chelated to Cr by fungi (Vale et al., 2016).

## 4 Conclusion

At present, the biosorption technology for metal adsorption is only at the laboratory level, and commonly used metal solutions are self-prepared at low concentrations (generally only a few tens of ppm). However, the concentrations of the heavy metal ions Ni, Co, and Re in the superalloy electrolyte used in this study were as high as 39088 mg/L, 10317 mg/L, and 2557 mg/L, with strong acidity. We pioneered the application of fungi, which are widely available and inexpensive, for adsorbing heavy metals from electrolytes with complex backgrounds and extremely high concentrations. Under optimal conditions, the adsorption rates of five precious heavy metal ions (Co, Cr, Mo, Re, and Ni) were 37.09, 64.41, 47.87, 41.59, and 25.38%, respectively. The adsorption of *P. lilacinus* on these five heavy metals followed the Langmuir and Freundlich adsorption isotherm models (*R*^2^ > 0.9), whereas the adsorption process of Cr was better fitted to the Freundlich adsorption isotherm model. This indicates that the adsorption of the five heavy metals was a complex bioaccumulation process that did not involve a single adsorption mechanism. The quasi-second-order model fitted well with the adsorption process of the five metals (*R*^2^ > 0.99), indicating that the rate-limiting step in the adsorption process was chemical adsorption. The theoretical maximum adsorption capacity obtained using second-order kinetics was similar to the experimental maximum adsorption capacity. Fourier transform infrared spectroscopy showed that the relevant active groups (hydroxyl, amino, amide, carbonyl, carboxyl, and phosphate) participated in the adsorption process.

It can be preliminarily concluded that *P. lilacinus* isolated in this study is an environmentally friendly, effective, and low-cost adsorbent with potential for electrolyte treatment. In the future, the combination of desorption and immobilization technologies could further improve the adsorption effect on electrolytes and more effectively recover heavy metals. At present, we are also actively exploring separation technologies for various metals in electrolytes, which is meaningful for alleviating metal resource scarcity and heavy metal pollution.

## Data availability statement

The original contributions presented in the study are included in the article/supplementary material, further inquiries can be directed to the corresponding authors.

## Author contributions

YY: Data curation, Formal Analysis, Investigation, Methodology, Project administration, Validation, Writing – original draft, Writing – review and editing. RL: Conceptualization, Methodology, Validation, Visualization, Writing – review and editing. YZ: Conceptualization, Resources, Writing – review and editing. YT: Conceptualization, Data curation, Formal Analysis, Methodology, Writing – review and editing. JZ: Methodology, Validation, Writing – review and editing, YW: Conceptualization, Investigation, Writing – review and editing. TD: Validation, Visualization, Writing – review and editing. PZ: Methodology, Writing – review and editing. XB: Writing – review and editing. SL: Data curation, Writing – review and editing.
